# Intra and Interobserver Reliability and Agreement of Semiquantitative Vertebral Fracture Assessment on Chest Computed Tomography

**DOI:** 10.1371/journal.pone.0071204

**Published:** 2013-08-05

**Authors:** Constantinus F. Buckens, Pim A. de Jong, Christian Mol, Eric Bakker, Hein P. Stallman, Willem P. Mali, Yolanda van der Graaf, Helena M. Verkooijen

**Affiliations:** 1 Julius Centre for Health Sciences and Primary Care, University Medical Centre Utrecht, Utrecht, The Netherlands; 2 Radiology Department, University Medical Centre Utrecht, Utrecht, The Netherlands; 3 Imaging Science Institute, University Medical Centre Utrecht, Utrecht, The Netherlands; Northwestern University Feinberg School of Medicine, United States of America

## Abstract

**Objectives:**

To evaluate the reliability of semiquantitative Vertebral Fracture Assessment (VFA) on chest Computed Tomography (CT).

**Methods:**

Four observers performed VFA twice upon sagittal reconstructions of 50 routine clinical chest CTs. Intra- and interobserver agreement (absolute agreement or 95% Limits of Agreement) and reliability (Cohen's kappa or intraclass correlation coefficient(ICC)) were calculated for the visual VFA measures (fracture present, worst fracture grade, cumulative fracture grade on patient level) and for percentage height loss of each fractured vertebra compared to the adjacent vertebrae.

**Results:**

Observers classified 24–38% patients as having at least one vertebral fracture, giving rise to kappa's of 0.73–0.84 (intraobserver) and 0.56–0.81 (interobserver). For worst fracture grade we found good intraobserver (76–88%) and interobserver (74–88%) agreement, and excellent reliability with square-weighted kappa's of 0.84–0.90 (intraobserver) and 0.84–0.94 (interobserver). For cumulative fracture grade the 95% Limits of Agreement were maximally ±1,99 (intraobserver) and ±2,69 (interobserver) and the reliability (ICC) varied from 0.84–0.94 (intraobserver) and 0.74–0.94 (interobserver). For percentage height-loss on a vertebral level the 95% Limits of Agreement were maximally ±11,75% (intraobserver) and ±12,53% (interobserver). The ICC was 0.59–0.90 (intraobserver) and 0.53–0–82 (interobserver). Further investigation is needed to evaluate the prognostic value of this approach.

**Conclusion:**

In conclusion, these results demonstrate acceptable reproducibility of VFA on CT.

## Introduction

Osteoporosis is a growing problem in the aging population, affecting up to one in three women and one in five men over 50 years of age [1], leading to millions of fractures annually and contributing substantially to morbidity and mortality [2], [3], particularly in the developed world. Subclinical vertebral fractures are an early sign of osseous fragility and their prevalence among adults is approximately 25%, increasing with age [4]. Subclinical vertebral fractures may precede overt osteoporosis and may predict future fractures, independently of dual-energy X-ray absorptiometry, which is currently the standard modality used to diagnose osteoporosis but has only modest predictive value for future fractures [5].

Vertebral fractures are visible on much routine clinical imaging that happens to visualize the spine, including chest Computed Tomography (CT). Despite being visible on chest CT, vertebral fractures are seldom assessed or reported unless this is specifically requested. Systematically reporting vertebral fractures and deformities on imaging that happens to visualize the spine would not require any additional imaging and could opportunistically identify patients who would benefit from preventative care. This is not currently common practice.

One of the most widely used methods for vertebral fracture assessment (VFA) is Genant's semiquantitative method [6], which assesses the shape of the deformity and its severity. Previously this method has been shown to have fair to good reproducibility and reliability on lateral CT scout views, radiographs or spinal densitometry [6]–[10]. Vertebral fractures may be even more readily detectable on CT than on conventional radiography [11]. To the best of our knowledge, the intra- and interobserver variability of vertebral fracture assessment of Genant's VFA method has not been studied on multislice CT. Knowledge on reproducibility and reliability is a necessary prerequisite for further investigations into the potentially substantial prognostic value of vertebral fractures on routine chest CT.

In this study, we determine the intra- and interobserver reliability and agreement of VFA on sagittal reformats of chest CT.

## Materials and Methods

Analysis and reporting of the study was performed according to the Guidelines for Reporting Reliability and Agreement Studies (GRRAS) [12].

### Source population and sampling

The present study was conducted in the context of the PROVIDI study, a study on the Prognostic Value of unrequested Information on Diagnostic Imaging. This multicenter study aims to establish the prognostic value of unrequested findings on thoracic CT and was described elsewhere [13]. Briefly, it includes all patients above forty years of age who underwent chest CT in one of eight participating Dutch hospitals between 2002 and 2005 (making it retrospective in nature), with exclusion of patients with a primarily oncological indication on radiological referral form [13]. As such it contains a heterogeneous range of protocols and reconstruction formats, representing routine practice. CTs from two academic centers and one peripheral center were deemed to be of sufficient quality to allow sagittal reconstruction. In the other hospitals the slice thicknesses of the stored CTs was >3-mm limiting for multi-planar reconstructions.

A random sample of 45 subjects was drawn from the available 6010 anonimyzed CT scans. The sample was ‘enriched’ with five subjects with moderate to severe vertebral fractures by a researcher who was not among the observers. The average age of the patients was 64 years (range: 54–79 years) and 34 (75%] patients were male.

### Vertebral fracture assessment

**Figure 1:**
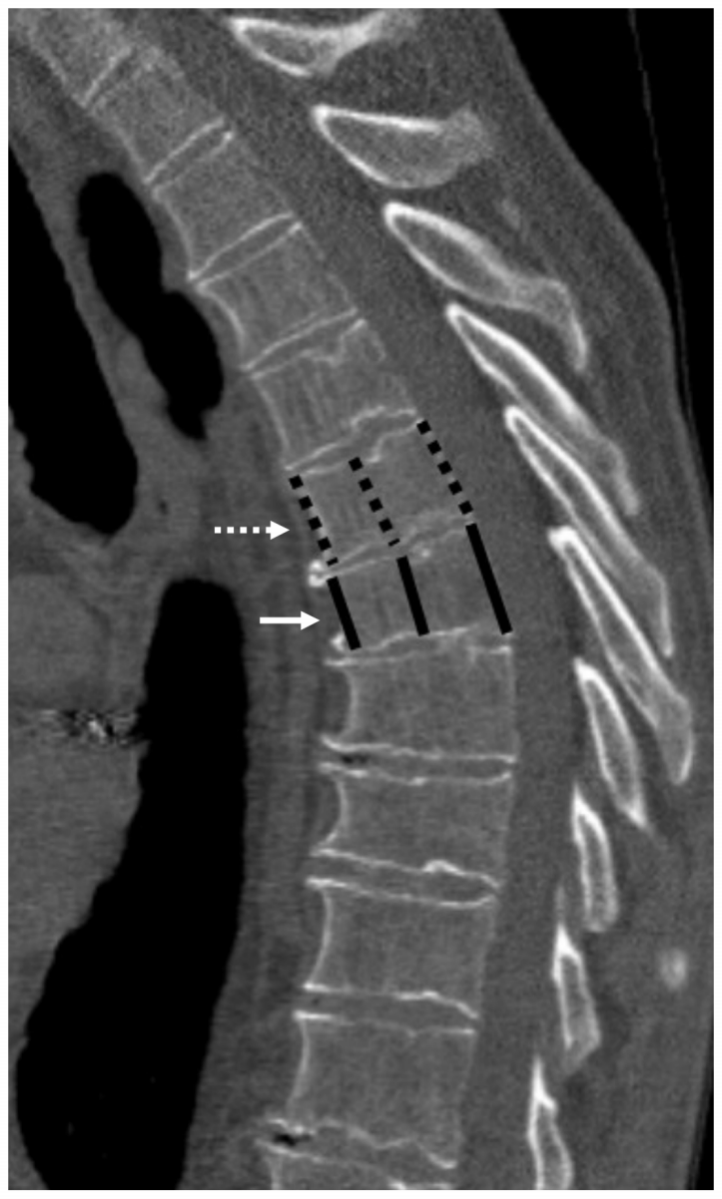
Moderate fracture. Degenerative spine showing a moderate (grade 2) wedge shaped fracture (solid arrow), with a reference vertebra immediately cranial (dashed arrow). Anterior, middle and posterior height measurement lines drawn on both. Measurements: For this patient, there is a fracture present, the worst fracture grade is 2, the cumulative fracture grade is 2 and the worst height loss of the fractured vertebra is the anterior height at 25%.

**Figure 2:**
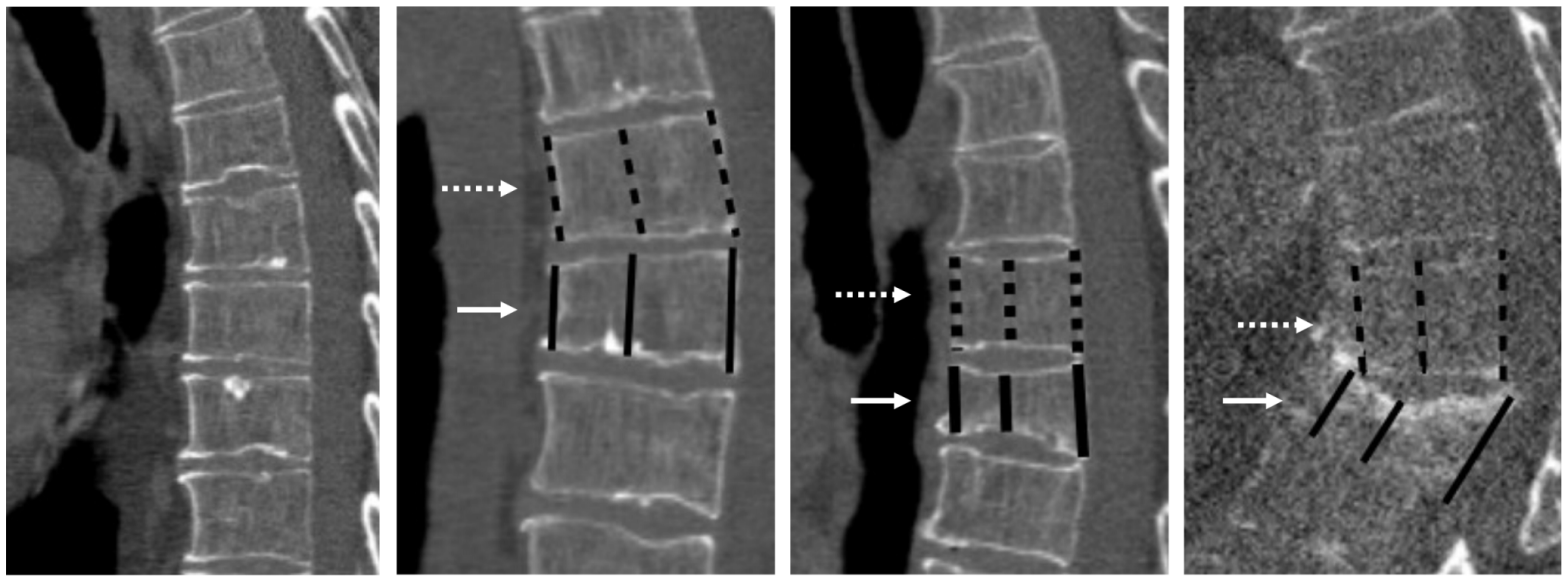
Sagittal reformats showing examples of all possible fracture stages. Panel a: Grade 0 (unfractured). Panel b: grade 1 (mild). Panel c: grade 2 (moderate). Panel d: Grade 3 (severe). Also shown are the anterior, middle and posterior height measurement calliper placements of the fractured vertebra (solid white arrows) and an adjacent reference vertebra (dashed white arrows).

Semiquantitative vertebral fracture assessment was performed by four observers with different levels of experience: one board certified chest radiologist with 10 years of experience, two radiology residents with 3 years and 4 years of experience and a research physician with less than one year of experience. For each individual patient, CTs were rated twice and in a different random order more than one week after the first VFA session. Raters received a brief introductory training prior to the first rating session. Observers assessed the vertebral body morphology of each visible vertebral body at or around the mid-sagittal slice for that level in bone settings (Figure 1). Observers recorded whether the visible vertebrae appeared to be fractured and graded the fractures according to Genant's semiquantitative VFA [6]. This method identifies and categorizes fractures according to the worst height loss relative to a normal unfractured vertebrae as height loss of 20–25% (mild), height loss of 25–40% (moderate) or height loss more than 40% (severe) (Figure 2).

In addition to the semiquantitative visual assessment we quantified the anterior, posterior and mid-body heights of the fractured vertebra and the adjacent normal vertebra using electronic calipers (Figure 1 and 2). Observers were instructed to use the vertebrae above (cranial to) the fractured vertebra as the ‘reference’ vertebrae when two equally distant vertebra were available (Figure 2). The height loss percentage was then calculated by taking the difference in the anterior, middle and posterior heights of the fractured and reference vertebra divided by the reference heights (and multiplying it by 100). For each fractured vertebra, the greatest percentage height loss (either anterior, middle or posterior) was used. The observers were not able to revise the subjective fracture grades based on these quantitative measurements.

### Analysis

Intra and interobserver agreement and reliability were estimated for five measures likely to hold prognostic relevance [14]: three patient-level measures (presence of a fracture, worst fracture grade and cumulative fracture grade) and two vertebral level measures (quantitative percentage height loss and presence of fracture) (Table 1). Note that the cumulative fracture grade, which is computed simply by summing up all fracture grades for each patient (i.e. two mild (grade 1) fractures and moderate (grade 2) one give a cumulative grade of four), is also known as the spinal deformity index [14].

**Table 1:** Outcome measures, their level of measurement (patient or vertebral), definition and the statistical methods applied to analyze intra- and interobserver agreement and reliability.

Level	Outcome	Definition	Measure	
			Agreement	Reliability
Patient	Fracture present	Fracture present (yes/no)	% absolute agreement	Cohen's kappa
	Worst fracture grade	Grade 0 = <20% height loss	% absolute agreement	Weighted kappa*
		Grade 1 = 20–25% height loss		
		Grade 2 = 25–40% height loss		
		Grade 3 = >40% height loss		
	Cumulative fracture grade	Sum of all grades for all fractures, continuous scale	95% Limits of Agreement	Intraclass Correlation Coefficient
Vertebral	Height loss	Measured height loss, expressed as percentage**	95% Limits of Agreement	Intraclass Correlation Coefficient
	Fracture present	Fracture present (yes/no)	% absolute agreement	Cohen's kappa

* Square weighted Cohen's Kappa.

** The fractured vertebra is compared to the nearest unfractured vertebra, with preference given to vertebrae cranial to (above) the fractured vertebra. The percentage of the worst height loss of each fractured vertebra (either anterior, middle or posterior part of the vertebral corpus), is given (see Figure 1).

On an intra- and an interobserver level, we assessed agreement and reliability (Table 1). Agreement indicates the absolute closeness of repeated measurements [15] and is particularly important when assessing the utility of a measure to track health status-changes over time using repeated measurements. For categorical measures (presence of fracture on both vertebral and patient levels, worst fracture grade) we computed absolute agreement [12] (i.e. the proportion of cases in which the first rating was exactly similar as the second). On the interobserver level, values were calculated for the first set of each observer only. Agreement of continuous measures (percentage height loss and cumulative fracture grade) was assessed using the Bland-Altman 95% limits of agreement [12], which can be interpreted as the maximum magnitude by which repeat measurements would be expected to differ in each direction, in 95% of repetitions. Reliability indicates whether a test can effectively distinguish between study objects (in our case either vertebrae or patients), despite observer error. The reliability of a measure is critically important in diagnostic practice, where distinguishing between affected and non-affected persons at a single time-point is the principle goal.

For the dichotomous measure (presence of a fracture on both patient and vertebral levels) we calculated Cohen's kappa's [12]. For the ordinal measure (the worst recorded fracture grade), reliability was assessed using square-weighted Cohen's kappa. Weighted kappa allows for the ordering in fracture grade assignment (mild – moderate – severe). Reliability is rated as ‘moderate’ for values between 0.41–0.60, as ‘substantial’ for values between 0.61–0.8 and as ‘excellent’ for values above 0.80 [16]. To investigate the reliability of continuous measurements (cumulative fracture grade and vertebral height loss) the Intra-Class Correlation Coefficient (ICC) was used. ICC's can be interpreted as the percentage of the variability between the ratings which is due to differences between the patients, and not due to observer error [12]. The two-way ICC(2,1) was computed for interobserver ICCs, to reflect the fact that a sample or patients and a sample of raters was observed, whilst a one-way ICC(1,1) was computed for the intra-observer ICCs.ICC's exceeding 0.7 are considered good and ICC's exceeding 0.8 excellent, with observer error having a negligible effect on observed correlations between two (sets of) measurements [17].

All analyses were performed using the R statistical software package (version 3.0.1 [18], with use of the ‘IRR’ package (version 0.83 [19]) for calculating the ICC's, kappa's and absolute agreement. For all reliability and agreement measures we present the values for the four observers as well as the ranges for the values observed. 95% confidence intervals were generated for the reliability measures using 2000 bootstrap replications.

### Ethics statement

This study was approved by the ethical review board of the University Medical Center Utrecht (decision number 06/193), which waived the need for written informed consent.

## Results

The observers scored between 12 and 19 (24 to 38%) of the included patients as having at least one vertebral fracture. The worst fracture grade observed was mild in 5 to11 patients, moderate in 2 to10 patients and severe in 2 to 6 patients. The median cumulative fracture grade for all four observers was 2 (range 0 to 14). Observers reported median height loss amongst the fractured vertebrae ranging between 29.3 to 35.6% (Table 2).

**Table 2:** Description of patient population: Frequencies and proportions or medians and ranges for each outcome based on first measurement session.

Level	Outcome		Observer 1	Observer 2	Observer 3	Observer 4
**Patient**	**Fracture present. N (%).**		15 (30%)	12 (24%)	16 (32%)	14 (28%)
	**Worst fracture grade. N (%).**	**Grade 0**	35 (70%)	38 (76%)	34 (68%)	36 (72%)
		**Grade 1**	5 (10%)	6 (12%)	8 (16%)	9 (18%)
		**Grade 2**	4 (8%)	2 (4%)	5 (10%)	2 (4%)
		**Grade 3**	6 (12%)	4 (8%)	3 (6%)	3 (6%)
	**Cumulative fracture grade. Median (range)** *		2 (1–14)	2 (1–8)	2 (1–13)	2 (1–9)
**Vertebral**	**Height loss (%). Median (range)** * **,** **		35.6 (3.2–72.3)	38.2 (19.3–74.2)	29.3 (6.1–79.1)	33.0 (4.5–72.3)
	**Fracture present. N (%).**		25 (3.7%)	16 (2.3%)	29 (4.3%)	24 (3.6%)

* Including only fractured vertebrae of patients classified as fractured.

** Note that some vertebrae, classified as fractured on visual assessment,showed an absolute height loss of less than 15% upon caliper measurement.

### Agreement

For patient-level fracture presence, the intraobserver agreement was between 88 and 94%, indicating that the observers classified the same patients similarly (i.e. unfractured or fractured) (Table 3). The interobserver agreement was lower, but still good, ranging from 82 to 92%. The worst fracture grade showed an intraobserver agreement of 76 to 88% and an interobserver agreement of 74 to 88%. For the cumulative fracture grade, the intraobserver and interoberserver 95% limits of agreement ranged from ±1.22 to ±1.99 and ±1.60 to ±2.69, respectively. This indicates that if the same or a different radiologist was to re-assess the same patient more than once, a change in fracture grade of 2 may be due to observer error alone but a change of 3 or more would be unlikely due to measurement error alone. The intraobserver and interobserver limits of agreement of the vertebral height loss ranged from ±5.97 to 11.75% and ±7.25 to 12.53%, respectively (Table 3). These values indicate that differences of up to 12.53% can be considered as measurement and observer error, upon repeat measurement of the same vertebra. The agreement for vertebral-level presence of fracture ranged from 97 to 99%, perhaps reflecting the low incidence of fractures on a vertebral level.

**Table 3:** Intra- and interobserver agreement for fracture presence, worst fracture grade, cumulative fracture grade and vertebral height loss.

Level	Outcome	measure	Agreement				
			Observer	1	2	3	4
**Patient**	**Fracture present**	**absolute agreement (%)** *	**1**	***88***	90	86	82
			**2**		***94***	84	84
			**3**			***90***	92
			**4**				***90***
	**Worst fracture grade**	**Absolute agreement (%)** *	**1**	***76***	82	78	74
			**2**		***88***	76	80
			**3**			***84***	88
			**4**				***84***
	**Cumulative fracture grade**	**95% Limits of Agreement** **	**1**	***±1.99***	±2.69	±1.6	±2.15
			**2**		***±1.8***	±2.58	±1.7
			**3**			***±1.8***	±1.84
			**4**				***±1.22***
**Vertebral**	**Height loss (%)**	**95% Limits of Agreement** **	**1**	***±5.97***	±7.25	±8.26	±11.71
			**2**		***±8.29***	±9.77	±11.31
			**3**			***±8.36***	±12.53
			**4**				***±11.75***
	**Fracture present**	**absolute agreement (%)** *	**1**	***98***	98	98	97
			**2**		***99***	98	97
			**3**			***98***	97
			**4**				***98***

Bold and italic = intra-observer.

* Percentage of absolute agreement in the first session of each observer for the interobserver and between the first and second sessions for the intraobserver.

** The 95% limits of agreement is the range of observer variation. This indicates that differences beyond this range cannot be ascribed to observer error alone.

### Reliability

For fracture presence the intraobserver reliability was good to excellent (kappa 0.73 (0.52–0.91) to 0.84 (0.63–1)) (Table 4). The interobserver kappa's ranged from 0.56 (0.29–0.79) to 0.81 (0.61–0.96), indicating fair to excellent interobserver reliability. For worst fracture grade, intraobserver reliability (weighted kappa) ranged from 0.84 (0.68–0.93) to 0.9 (0.78–0.96) whilst the interobserver scores ranged from 0.73 (0.45–0.88) to 0.88 (0.67–0.97), indicating substantial to very good reliability. For the cumulative fracture grade, the intraobserver reliability was excellent (ICC's: 0.84 (0.71–0.94) to 0.94 (0.65–0.98)), and the interobserver was good to excellent (0.74 (0.57–0.93) to 0.94 (0.57–0.98)). The interobserver reliability of vertebral height loss was moderate to good (ICC: 0.59 (0.33–0.77) to 0.9 (0.81–0.95)), as was the interobserver (ICC: 0.53 (0.21–0.73) to 0.82 (0.62–0.92)). This rather large range was attributable to one of the four observers, without which the intra- and interobserver minimums would have been 0.75 (0.48–0.91) and 0.7 (0.42–0.85), respectively. A similar pattern repeated itself in the presence of fracture measure on a vertebral level: the intra-observer reliability ranged from 0.56 (0.38–0.71) to 0.74 (0.55–0.88) and the inter-observer reliability ranged from 0.39 (0.18–0.58) to 0.63 (0.43–0.79).

**Table 4:** Intra- and interobserver reliability for fracture presence, worst fracture grade, cumulative fracture grade and vertebral height loss.

Level	Outcome	Measure	Reliability				
			Observer	1	2	3	4
**Patient**	**Fracture present**	**Kappa**	**1**	***0.73 (0.52–0.91)***	0.75 (0.50–91)	0.67 (0.42–0.88)	0.56 (0.29–0.79)
			**2**		***0.84 (0.63–1)***	0.61 (0.34–0.83)	0.59 (0.29–0.82)
			**3**			***0.78 (0.58–0.96)***	0.81 (0.61–0.96)
			**4**				***0.76 (0.52–0.95)***
**Patient**	**Worst fracture grade** *	**Weighted kappa** *	**1**	***0.84 (0.68–0.93)***	0.85 (0.67–0.95)	0.79 (0.56–0.92)	0.73 (0.45–0.88)
			**2**		***0.89 (0.75–0.98)***	0.82 (0.58–0.92)	0.87 (0.65–0.94)
			**3**			***0.9 (0.78–0.96)***	0.88 (0.67–0.97)
			**4**				***0.89 (0.71–0.96)***
**Patient**	**Cumulative fracture grade**	**ICC** **	**1**	***0.91 (0.54–0.97)***	0.75 (0.61–0.96)	0.94 (0.57–0.98)	0.87 (0.45–0.93)
			**2**		***0.84 (0.71–0.94)***	0.74 (0.57–0.93)	0.87 (0.48–0.96)
			**3**			***0.91 (0.76–0.95)***	0.89 (0.56–0.94)
			**4**				***0.94 (0.65–0.98)***
**Vertebral**	**Height loss (%)**	**ICC** **	**1**	***0.9 (0.81–0.95)***	0.82 (0.62–0.92)	0.81 (0.65–0.90)	0.56 (0.27–0.75)
			**2**		***0.75 (0.48–0.91)***	0.7 (0.42–0.85)	0.53 (0.21–0.73)
			**3**			***0.82 (0.68–0.90)***	0.55 (0.29–0.73)
			**4**				***0.59 (0.33–0.77)***
**Vertebral**	**Fracture present**	**Kappa**	**1**	***0.72 (0.58–0.85)***	0.63 (0.43–0.79)	0.58 (0.41–0.73)	0.43 (0.24–0.59)
			**2**		***0.74 (0.55–0.88)***	0.57 (0.37–0.73)	0.39 (0.18–0.58)
			**3**			***0.71 (0.57–0.82)***	0.51 (0.32–0.67)
			**4**				***0.56 (0.38–0.71)***

Bold and italic = intra-observer.

* Square-weighted kappa.

** The Intraclass Correlation Coefficient (ICC).

(95% confidence intervals based on 2000 bootstrap replicates).

## Discussion

Vertebral fracture assessment on routine chest CT scans in an adult population shows generally good reliability and agreement. Specifically, for fracture presence and worst fracture grade we found excellent reliability and agreement. For cumulative fracture grade we found good reliability but modest agreement. For vertebral height loss we found good agreement but modest reliability, largely attributable to one of the observers.

Reliability indicates the ability of a test to distinguish between different individuals in spite of measurement error, whilst agreement indicates the absolute closeness of repeated measurements. For example, a weighing scale may be able to accurately and reproducibly measure the body weight of patients with a low margin of error, thus having good agreement. The reliability however also depends in part on the variability of the body weight between the patient sample of interest. If they have body weights very close together (low variability), even the scale's small margin of error will confound its reliability and the reliability values associated with it will be low.

Our findings are in line with the reported interobserver reliability for the semiquantitative method on conventional radiography (interobserver kappa values ranging from 0.60 to 0.80 [6], [9]) and demonstrate that semiquantitative vertebral fracture assessment method can reliably be applied on sagittal reconstructions of chest CTs. The participating observers represent a range of of different levels of radiological experience, including a relative novice with less than one year of experience, two intermediate observers with several years of experience each and a highly experienced board certified radiologist. This range is representative of clinical practice.

Since the majority of vertebral fractures are clinically silent and underreported, the diagnosis is often delayed. Presumably this underreporting is due to a number of reasons, including the extra time involved in creating and assessing the necessary sagittal reformats, the tendency of radiologists to focus on requested pathologies, unfamiliarity with the application of vertebral fracture assessment to CT and a general uncertainty surrounding the prognostic implications of subclinical vertebral fractures. By showing the reliability of well-established vertebral fracture assessment schemes on sagittal CT, the willingness to consider vertebral fracture assessment on CTs may increase. The detection of subclinical vertebral fractures on routine imaging that happens to visualize the spine has the potential to be a useful and cost-effective means of identifying patients at risk for future osteoporotic fractures, who can then be treated preventatively with fall prevention, lifestyle advice, hormonal supplementation and mainly antiresorptive medication; interventions that are proven to reduce fracture risk. There is growing momentum to this end; current guidelines already list these fractures as an indication for treatment [20].

For cumulative fracture grade, the modest limits of agreements we found may be acceptable in practice. A previous study [14] has shown that the cumulative fracture grade is predictive for future fracture risk, particularly when the grade >3 and especially when >7. Therefore the maximal limits of ±2.69 do not necessarily preclude the prognostic utility of this measure. However, further research is needed to determine which cut-offs are most prognostically useful.

The vertebral level height loss measurement performed additionally to the standard visual assessment as proposed by Genant showed limits of agreement very close to the minimum height difference which a trained observer is likely able to detect (i.e., 13% [21]). The reliability values (ICC's) however varied widely across the observers. This suggests that the reliability of this VFA measure may also fluctuate similarly in clinical practice. This variability was also repeated for the vertebral level presence of fracture (although not the patient-level presence of any fracture variable). Furthermore, some low (>20%) height loss values were found in vertebrae that were classified as (usually mildly) fractured upon visual inspection. This may be due to unfractured vertebrae being misclassified as fractured and/or due to incorrect calliper measurement. These problems that observers had with quantification, which was also the most time-consuming part of the study, may be overcome by automated vertebral body height measurement on CT in the future.

### Limitations

Since PROVIDI scans were acquired and stored between 2002–2005, and were retrospectively reconstructed, prospective reconstruction with new scanner generations would presumably result in better image quality and in theory non-comparability to our findings. Whilst a prospective study with the attendant better quality of stored reconstructions could result in better reliability and agreement, we feel our dataset provides a realistic assessment for how VFA might perform across a spectrum of scanner generations currently in use in a variety of settings and locales.

Inherent to our study design, we lack an external reference standard with which to compare the observer's ratings. Additional imaging performed in PROVIDI patients, such as lateral chest X-rays, which might have been used for this purpose, were not included in the original study design and are also beyond the scope of this paper. Demonstrating the reliability and agreement does not require such an external ‘gold standard’ as comparisons are done between and within observers, rather than with an external reference standard, as in a diagnostic accuracy study. We enriched our sample to ensure an adequate number of higher fracture severities would be present. We believe that this is unlikely to have influenced agreement [22] and reliability measures, as previous studies investigating the prevalence of vertebral fractures on routine clinical CT showed prevalence of vertebral fractures similar to ours, ranging from 10–35% [23]–[26]. Finally, the clinical histories of the included patients were not available within the PROVIDI cohort. Consequently other causes of vertebral fracture such as past major trauma were not known, nor was it known which proportion of the patients identified with fractures were already receiving fracture prevention. Prior literature suggests that only a minority of fractures will have been known and a minority will have been under treatment [27].

In conclusion, we found that semiquantitave vertebral fracture assessment can be applied on standard sagittal reconstructions of routine clinical chest CTs with acceptable reliability and agreement. Future research to evaluate the prognostic value of these VFA measures on routine clinical CTs should elucidate which of the four VFA is the strongest predictor of future fractures.
